# Formant analysis of vertebrate vocalizations: achievements, pitfalls, and promises

**DOI:** 10.1186/s12915-025-02188-w

**Published:** 2025-04-07

**Authors:** W. Tecumseh Fitch, Andrey Anikin, Katarzyna Pisanski, Daria Valente, David Reby

**Affiliations:** 1 Department of Behavioral and Cognitive Biology, University of Vienna, Djerassiplatz 1, Vienna, 1030 Austria; 2 Division of Cognitive Science, Lund University, Helgonavägen 3, Lund, 223 62 Sweden; 3 ENES Bioacoustics Research Lab/Lyon Neuroscience Research Centre (CRNL), University of Saint-Etienne, CNRS UMR5292, INSERM UMR_S 1028, 21 rue du Dr. Paul Michelon, Saint-Étienne, 42100 France; 4 CNRS French National Centre for Scientific Research, DDL Dynamics of Language Lab, University of Lyon 2, Lyon, 69007 France; 5 Institute of Psychology, University of Wrocław, Poland, Jana Władysława Dawida 1, Wrocław, 50-529 Poland; 6 Department of Life Sciences and Systems Biology, University of Torino, Via Accademia Albertina 13, Torino, 10123 Italy; 7 Institut Universitaire de France, 1 Rue Descartes, Paris, France

**Keywords:** Vocalization, Animal communication, Evolution of communication, Vocal production, Formant frequency, Source-filter theory

## Abstract

When applied to vertebrate vocalizations, source-filter theory, initially developed for human speech, has revolutionized our understanding of animal communication, resulting in major insights into the form and function of animal sounds. However, animal calls and human nonverbal vocalizations can differ qualitatively from human speech, often having more chaotic and higher-frequency sources, making formant measurement challenging. We review the considerable achievements of the “formant revolution” in animal vocal communication research, then highlight several important methodological problems in formant analysis. We offer concrete recommendations for effectively applying source-filter theory to non-speech vocalizations and discuss promising avenues for future research in this area.

**Brief** Formants (vocal tract resonances) play key roles in animal communication, offering researchers exciting promise but also potential pitfalls.

## Introduction

Formants— resonances in the vibrating air of the vocal tract —amplify specific frequencies during voice production. Formants play a central role in the acoustics of human speech, and human formants have thus been subject to intensive study over the past fifty years. More recently, the last two decades have witnessed an explosion of interest and research on formants in nonhuman animal vocalizations. Peaks in the vocal frequency spectrum corresponding to vocal tract resonances have now been demonstrated in many clades: reptiles, birds, and mammals including ruminants, marsupials, carnivores, and nonhuman primates. Furthermore, playback studies show that conspecifics in all these clades perceptually attend to formants. Thus, far from being specific to human speech, formants exist and are perceived in a wide range of vertebrates, strongly suggesting that they represent a basal feature of vocalizations observed in most extant amniotes [1, 2].

Although researchers have only recently begun to explore the communicative functions of formants in vertebrate vocalization, it is already clear that formants often provide acoustic cues to identity [3], body size [4], and affective states [5], and that they can do this independently of other potential acoustic cues such as voice pitch [6] (see [7, 8] for reviews). For example, formant-based cues to body size can play a key role in mating decisions and dominance contests, thus having important effects on fitness. Unsurprisingly, numerous species have independently evolved physiological and anatomical “tricks” aimed at adjusting formants in order to maximize the impression of large body size conveyed by vocalizations [9, 10]. These include laryngeal lowering in multiple species including humans, tracheal elongation in many clades of birds, and laryngeal air sacs in many mammals (including all great apes). In summary, the study of formant frequencies in animal vocalizations has established formants as widespread and salient acoustic phenomena that play important roles in social and sexual interactions and has shown that they have driven the convergent evolution of fascinating vocal tract morphological features whose functions had previously remained mysterious.

Despite its promise to provide a deeper understanding of the way distinct sound components combine and contribute to vocal communication, the study of formants is not without pitfalls. Because formants are a second-order cue that involves filtering some pre-existing source sound, they depend upon an appropriate source to be detectable—ideally including broadband noise or a relatively dense harmonic stack resulting from a relatively low fundamental frequency (see Table 1 for definitions of key terminology). If an appropriate source signal is absent, accurately measuring formants can be difficult or even impossible in some vocalizations. Furthermore, there are several classes of “pseudo-formants” that produce spectral peaks reminiscent of formants that do not, in fact, correspond to vocal tract resonances. Finally, even after formants have been accurately detected and measured, open questions about the underlying physics of distinct species’ vocal production can complicate inferences about the crucial underlying variables that are perceptually and biologically relevant.

**Table 1:** Definitions of key terminology

Glossary	
Formant	A vocal tract resonance and/or a prominent spectral peak resulting from such a resonance. The relative positions of the first three formant frequencies (*F*1 to *F*3) determine the vowel quality in speech, whereas formant spacing among all measurable formants can be used to estimate apparent vocal tract length.
Fundamental frequency (*f*_o_)	The lowest frequency at which a periodic signal is repeated. For voiced signals in most tetrapods, *f*_o_ corresponds to the rate at which the vocal folds are vibrating and is the perceptual correlate of pitch.
Nonlinear acoustic phenomena	Deviations from regular phonation such as frequency jumps (sudden changes in *f*_o_), sidebands (amplitude or frequency modulation of the glottal source by additional oscillators), subharmonics (irregular vibration of the vocal folds, which produce weaker secondary frequencies at an integer fraction of *f*_o_), and deterministic chaos (non-periodic vibration of the vocal folds).
Pitch	The perceived height or musical tone of a sound. In voiced signals, pitch is mainly determined by the fundamental frequency.
Resonances	In physics, the natural frequencies of an oscillator at which an external driving force produces maximum response. In voice science, this often refers to the frequencies that are preferentially transmitted by the air in the vocal tract.
Vocal control	The capacity to control the larynx (affecting the production of *f*_o_) or the vocal tract (affecting the production of formants) in a flexible and/or voluntary manner, for example as a function of social context.
Vocal tract length (VTL)	The length of the airway from the sound-producing source to the aperture through which the sound is radiated into the environment (e.g., the mouth, nostrils, or beak). Formant frequencies scale inversely with VTL, so elongating the vocal tract by 10% on average lowers formants by 10%.
Voice modulation	Dynamic (time-varying) change of any property of the voice including but not limited to *f*_o_ and formant frequencies.
Vowel quality	Formant frequencies are equally spaced in a cylindrical vocal tract, which approximately corresponds to the neutral *schwa* vowel /ә/ (all phonetic symbols are taken from the International Phonetic Alphabet). When articulatory movements change the shape of the vocal tract, the lower formant frequencies are modified from these rest positions, and different vowels are produced. Because absolute formant frequencies depend on vocal tract length, and thus vary across speakers, vowel quality is best operationalized as speaker-normalized *F*1 and *F*2 relative to schwa.

In this paper, we first briefly review the acoustic origin and physical nature of formants and discuss the exciting results of recent bioacoustic research on formants in vertebrate vocal behavior. We cover basic source-filter theory, the essential lack of coupling between source and filter—and how to test this with heliox experiments—along with vocal body size allometry, formant perception, and the size-exaggeration hypothesis for anatomical augmentations of the vocal tract. This concise review constitutes the *achievements* component of our paper.

We then turn to a more detailed consideration of the challenges and potential *pitfalls* of formant research in animal communication, explaining the issues and illustrating them with both real and synthetic examples, and referring to recent published studies that appear to fall into these traps. Our goal is not to discredit or shame such work, but to illustrate the reality of these pitfalls and offer constructive suggestions to help avoid them in future research. Specifically, we discuss the issues of source under-sampling, measuring harmonics instead of formants, sidebands caused by amplitude or frequency modulation but sometimes mistaken for formants, and chaotic pseudo-formants that are properties of the source (vocal fold vibrations) rather than the filter (vocal tract).

To help researchers avoid these pitfalls, we describe a statistical framework for evaluating formants that uses a set of accurately measured formant frequencies as input and produces one or more “compressed” parameters as output, that together offer potential quantitative proxies for the perceptual inferences made by conspecific listeners. In its simplest form, this modeling framework provides a single value, corresponding acoustically to normalized formant spacing and physiologically to vocal tract length, assuming that the vocal tract is a simple uniform tube anatomically. This regression-based framework also allows deviations from the simple tube idealization to be quantified and evaluated and provides a powerful method to analyze vowel-like patterns of formant deviation normalized for overall body size. Our analysis of vocal formants from 13 vertebrate species using this method shows that nonhuman species explore a significant portion of the so-called vowel space well-known in human speech, further calling into question the human exceptionalism that has traditionally characterized speech science.

In the final *promises* section, we conclude by highlighting numerous exciting open questions and testable hypotheses that remain unresolved or even unexamined.

### The source-filter theory of vocal production

**Fig. 1:**
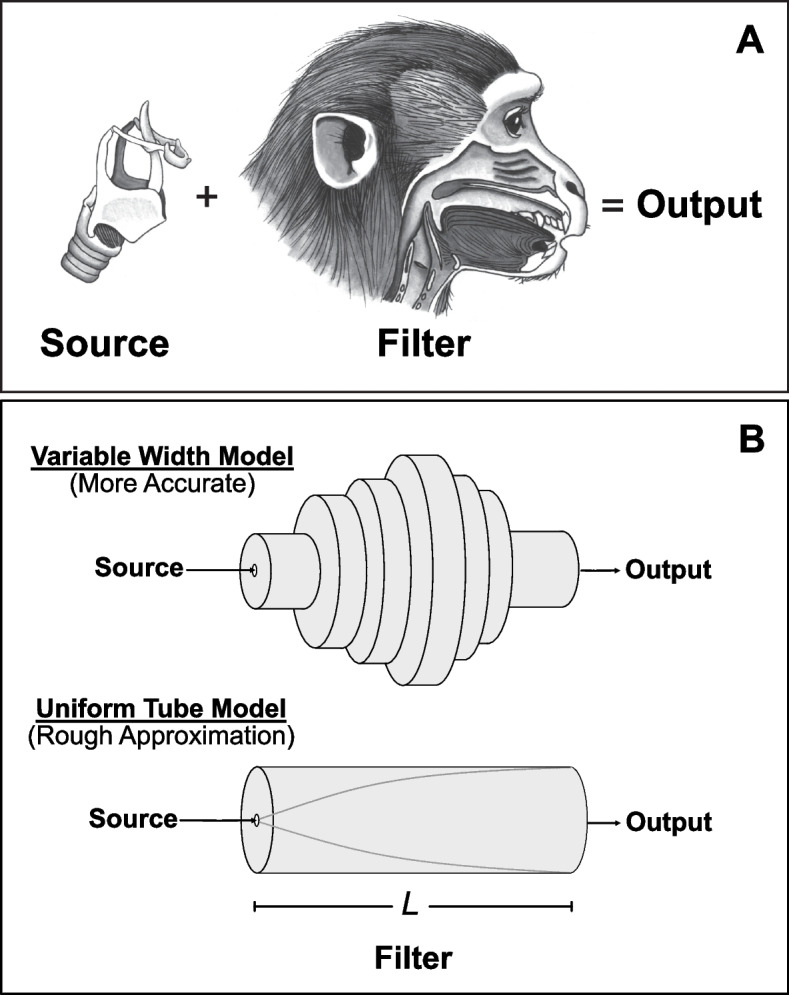
Basic source-filter theory.** A** A schematic larynx and vocal tract of a nonhuman primate, illustrating that the sound output from the vocal tract combines features of the source (typically generated by vibrating vocal folds within the larynx) and the vocal tract filter. **B** Two ways to model the vocal tract. In the upper schematic (“Variable Width model”), the variability in diameter of the vocal tract as we ascend from source to output is modeled as a series of variable-width “tubelets.” In the lower schematic (“Uniform Tube model”), a simple tube of uniform width down its entire length is used. In both cases, the length *L* of the filter should match the length of the anatomical vocal tract being modeled. The latter, simplified model allows an easy calculation of predicted formant frequencies (see main text)

The central conceptual framework for understanding vocal production in vertebrates is the source-filter theory. This theory was originally formulated for human speech [11, 12] and singing [13], but has since been extended to many other vertebrates from deer [6] and elephant seals [14] to cranes [15], penguins [16], alligators [17, 18], and marmosets [19]. Source-filter theory is conceptually simple: an organism’s vocal output is a combination of two independent physical systems: the source and the filter (Fig. 1). Although there is considerably more published research on formants in mammals, particularly primates, the principles of source-filter theory clearly apply to most other terrestrial vertebrates including birds [15, 20–24], frogs [25] and reptiles [17, 18].

Sound is initially generated in the source organ. Typically this is the larynx in mammals, reptiles and amphibians, and the syrinx in birds. In the source, a silent, pressurized flow of air from the lungs induces small pieces of tissue (the vocal folds in humans and other mammals, and syringeal tissues in birds) to vibrate and collide with one another. These movements cause the opening between the vocal folds, termed the glottis, to open and close rapidly, releasing regular puffs of air. This causes pressure pulses that propagate further as sound and constitute the source signal. When the tissue oscillation is periodic, as in singing or most speech, the rate of vocal fold vibration is termed the fundamental frequency (*f*_o_) and it is the physical correlate of perceived pitch.

Because this periodic source signal is not a pure sinusoid, it has a complex spectrum with energy not only at the fundamental frequency *f*_o_ but also at integer multiples of this frequency: 2*f*_o_, 3*f*_o_, 4*f*_o_, etc. Thus, a fundamental at 100 Hz would yield harmonics at 200, 300, 400 Hz, etc. The source signal therefore has energy at many specific frequencies, but the spacing between the harmonics (their density) is entirely determined by the fundamental frequency *f*_o_. In addition to this periodic component, the laryngeal vocal source typically includes aperiodic noise created by air turbulence in the glottis and is clearly audible in breathy speech and in consonants like [h], or even while simply breathing with an open mouth. This entire “bouquet” of frequencies emitted by the source organ now enters the vocal tract for further processing.

Turning to the filter component, the vocal tract consists of the respiratory passages connecting the source organ to the outside environment, including the throat (pharynx), the oral cavity, and the nasal cavities in all tetrapods. Additionally, in birds, the vocal tract includes the trachea because the syringeal source is located at the base of the trachea. These connected tubes of air together make up the vocal tract, and their shape and configuration can be modified in various ways. Crucially, the air within the vocal tract can vibrate at multiple resonance frequencies termed *formants*. These formants act as a filter, shaping the initial source signal by preferentially transmitting source energy that aligns with formant frequencies while suppressing energy that lies between them. The vocal tract filter thus shapes or “sculpts” the source signal, and the output sound that we hear (or record with a microphone) is a linear combination of the source and filter.

Unlike the harmonics of the source signal, the formants that make up the filter are relatively independent of one another, and their frequencies can be modified by various articulatory maneuvers: rounding or spreading the lips, moving the tongue in the mouth and throat cavities, raising the velum or soft palate to close off the nasal passages, etc. [13]. It is precisely such changes in formant frequencies that determine the different vowels in human speech [14, 26]. Despite this relative freedom of individual formant frequencies to vary (compared to harmonics), the entire series of formants is constrained by the overall length of the vocal tract: longer vocal tracts produce lower and more densely spaced formants than do shorter vocal tracts in both humans [27, 28] and nonhuman animals [10]. In general, we can roughly approximate the formants expected for a vocal tract of a given length using the following equation:

1 $$\large F_i\;=\;\frac{nc}{4L}; i=\;1,\;2,\;3,\;4...,\;n\;=\;1,\;3,\;5,\;7...$$

where *c* is the speed of sound in warm humid air (~ 350 m/s), *L* is the length of the tube, and *F*_*i*_ denotes the frequency of the successive *i*th formant corresponding to the odd *n* multiples of a quarter wavelength resonator. That is, the lowest formant will be a quarter wavelength, the next formant *F*2 at 3/4 wavelength, etc. Importantly, this equation assumes that the vocal tract is a simple uniform tube open at one end (the mouth) and closed at the other (the glottis). In fact, the vocal tract will very rarely have a completely uniform area along its length, and deviations from uniformity will cause corresponding deviations in formant frequencies (see Fig. 1). Furthermore, the “half open” assumption may not always be true, although it appears to apply to many mammals [8] along with both crocodilians and birds [17, 18, 24]. For a more detailed discussion and explication see ( [13]: p.156).

Turning to perception, key aspects of both the source and filter are perceived by the vertebrate auditory system. Regarding the source and fundamental frequency, the best understood percept is that of “pitch”—the perceived degree of highness or lowness of a tonal sound. Our perception of voice pitch is closely tied to the fundamental frequency of vocal fold vibration *f*_o_, but they are not the same thing: *f*_o_ is an objective property of the physical system, while pitch is a subjective psychological variable, inferred by the listener from the acoustic signal. Thus, if a wire vibrates in the woods and no one hears it, it has an *f*_o_ but does not have a pitch. Although pitch perception scales logarithmically, not linearly, with *f*_o_, the perceived pitch is typically tied closely to *f*_o_ in the frequency range of the adult human voice during modal speech production (around 80–300 Hz). Nevertheless, under certain circumstances, they differ. For example, so-called missing fundamental stimuli have no energy at *f*_o_, but do have energy at higher harmonics 2*f*_o_, 3*f*_o_, and 4*f*_o_. In this situation (for example, when a low-frequency sound is transmitted on a bad phone line or small loudspeaker), our percept of the pitch still corresponds to the missing *f*_o_ despite there being no energy present at that frequency. Bandpass-filtered aperiodic noise also evokes a salient sensation of pitch, which makes it clear that we must distinguish properties of the underlying physical system from both the acoustic signal and our perception of that signal and system. Often, there is a reasonably clean, direct correspondence between each link of this three-part chain, but this is not always true. Thus, although it is common to speak of “voice pitch” as equivalent to *f*_o_, strictly speaking, this is incorrect. This three-way distinction will play an important part in the rest of our discussion.

Regarding formant perception, and unlike pitch perception, there is no standard English word to distinguish the ‘physical’ properties of formants (as measurable components of the biophysical production system) from the ‘perceptual’ properties of formants (as perceived by the ear), so researchers often use the same term for both sides of this coin. Perceptually, changes in formant frequencies lead to changes in "timbre", with low formants leading to a “darker”, more baritone timbre, and high formants leading to a squeakier, more childlike timbre. This is reminiscent of the tonal change when playing the same note on a cello versus a violin: the *f*_o_ is the same, but the resonant frequencies are lower on the cello due to its larger body (the "filter") so they still sound distinctly different.

### Formant frequencies

Formant (*for – mәnt*; from Latin *formare*, “to shape”).

We can define a formant as: (1) a resonance of the vocal tract; (2) a peak in the spectrum of a vocal signal resulting from a vocal tract resonance; (3) the perceptual correlate of a spectral peak caused by a vocal tract resonance. All three of these definitions are used in voice science, but the first is most common and is adopted here.

The term “formant” was introduced as a key component of speech by Ludimar Hermann in 1894 [29] and rapidly adopted by speech scientists [30, 31]. The central importance of formants to speech became widely realized in the mid-twentieth century with the crystallization of source-filter theory, marking a major breakthrough in the study of vowel and consonant production and perception [11, 12]. Linguists and phoneticians quickly came to realize the central role of formant frequencies in creating phonetic diversity in human speech. Most notably, the relative spacing of the lower formants encodes specific vowel sounds (vowel quality) in a similar manner across languages (International Phonetic Alphabet). For instance, the closed-front vowel /i/ (as in “beet”) is characterized by a wide gap between formants *F*1 and *F*2, while the open-back rounded vowel /ɒ/ (“bought”) is characterized by a relatively small gap between these same lower formants [32]. Transitions in formant spacing are effortlessly and rapidly achieved during typical speech production by manipulating the lips, tongue, and jaw, and thus the overall dimensions of the oral cavity. An /u/ vowel sound (“boot”), for example, can be achieved by bunching the tongue and rounding the lips, which constricts the anterior oral cavity [32]. The early study of formants in speech also clarified how consonants are encoded in formant transitions, resulting in coarticulation, a revolutionary discovery in phonetics [33].

In the modal speech of adult humans, formant perception profits from a relatively low and stable fundamental frequency resulting in a dense harmonic structure that facilitates formant perceptual salience and thus functionality. These dense harmonics in speech, like a densely pixeled high-resolution photograph, aid both vowel perception [34] and body size perception from the human voice [35] because both identity and size perception rely largely on the discernment of formants [28]. Despite this crucial relationship between *f*_o_ and formant frequency measurement or perception (see also [36]), a critical tenant of source-filter theory is the relative independence of source and filter frequencies. In speech, vocal fold and vocal tract dynamics are typically decoupled and can vary freely, independently of one another (Table 1).

Second-order interactions between source and filter are known to occur, for example in singing with a falsetto voice when *f*_o_ approaches a formant frequency [37, 38], and a formant may further destabilize an already unstable source leading to voice breaks, but these are secondary effects in the human voice [39] and their relevance to nonhuman bioacoustics remains unclear.

Comparing voices in atmospheric conditions of heliox versus ambient air provides a powerful method to test for source-filter independence. The source-filter system involves independent tissue vibrations of the source and air vibrations in the filter. When source and filter are uncoupled, filling the respiratory system with a different gas, helium, which is less dense than air and conveys sound more rapidly, will cause the formant frequencies to shift uniformly upwards while leaving tissue-based source frequencies (*f*_o_ and its harmonics) unchanged. Because the wavelength $\lambda$ of the formant (determined by vocal tract dimensions, e.g., using Eq. 1) remains the same, the change in frequency *F* will depend only on the change in the speed of sound *c* according to a simple equation:

2 $$F\;=\;c/\lambda$$

For living organisms, a mixture of helium and 20% oxygen (“heliox”) is used to allow normal respiration during such experiments. Vocalizations in heliox, which sound uncannily high in timbre, were first used to demonstrate independence of source and filter in humans in the early 1960s [40]. Helium chambers have since been used to show source-filter decoupling in many other animals, from songbirds [21, 22], frogs [25], and alligators [17], to bats [41], dolphins [42], and nonhuman primates [19, 43].

Interestingly, an early study utilizing the heliox method with a nonhuman animal was conducted on the California sea lion [44]. At the time of its publication in the mid-60 s, the study’s results were not interpreted within the source-filter framework, which had not yet made its mark in bioacoustics. In light of what we now know, Brauer and Jennings’ early work constitutes some of the first evidence of formants in a marine mammal.

A known biological exception to source-filter independence is provided by the ultrasonic whistles produced by mice, rats, and various other rodents, or by human lip whistling [45]. The source of sound in an aerodynamic whistle is oscillations in the gas itself, channeled by the whistle’s static geometry, but governed by purely aerodynamic forces [45, 46]. Thus *f*_o_ in whistles is determined by the rate of vortex shedding in the gas and is not created by tissue vibrations. To produce a steady pitch, the rate of vortex shedding must be stabilized by a coupled resonator, which in the case of human lip whistling is the oral cavity. In this case, the resonances of the vocal tract are strongly coupled to, and determine, the *f*_o_ of the whistle [45]. Similarly, because the source in rodent aerodynamic whistles is coupled to vocal tract resonances, rodents in heliox produce ultrasonic calls with shifted fundamental frequencies [47, 48], indicating the inapplicability of standard source-filter theory to these specific sound types.

### Achievements

Although the difficulty with which nonhuman primates can control their formants relative to humans was already discussed in the late 1960s [49, 50], it was not until the late 1980s and early 1990s that the relevance of formants to nonhuman animal vocal communication really began to be realized, first in cat vocal production [51, 52]. Research in this area then took off at the turn of the twenty-first century [3, 4, 20, 53]. Source-filter theory radically changed the face of bioacoustics once it became widely applied to animal calls, providing a solid theoretical framework within comparative bioacoustics to test predictions about form and function in animal communication, while also looking for precursors of human speech-like abilities.

Early influential work linked formants to vocal tract length and thus body size [53], with mounting comparative evidence now showing that formants, whose overall spacing is constrained by vocal tract length, are among the most reliable acoustic predictors of an animal’s size, even when controlling for sex and age. This is because overall formant spacing scales inversely and allometrically with vocal tract length in terrestrial mammals, and the vocal tract grows proportionately to the rest of the body. The first evidence of a formant-size relationship came from rhesus macaques [53], quickly followed by an accumulation of converging evidence from dozens of other mammalian species (for reviews see [7, 8, 54]).

Playback experiments of resynthesized male sexual loud calls to red deer stags during the reproductive period have further confirmed that harem-holding stags respond more aggressively to roars in which lower formants mimic larger opponents [6]. Similarly, when in oestrus, female red deer prefer roars where formants have been re-synthesized to mimic larger stags over those of smaller stags [36]. Together the results of these playback experiments strongly suggest that, at least in some species, formant frequencies are perceived as cues to body size in both competitive and mate choice contexts.

In humans, formant spacing explains several times more variance in height (when age and sex are controlled for) than does fundamental frequency *f*_o_, which does not robustly predict men’s or women’s heights within sexes [28]. While human listeners perceptually associate both low *f*_o_ and low formants with large body sizes, they prioritize information from formants when the two frequency parameters are manipulated to be equally perceptually salient [55].

Research on formants in animal calls also led to critical discoveries regarding their role in deceptive signaling. Despite anatomical constraints that impose some degree of honesty on formants as reliable cues to body size, selective pressure for size exaggeration has led to the evolution of anatomical adaptations of the vocal apparatus in a diverse range of species. For example, the presence of descended and mobile larynges in the males of several mammal species can now be explained in terms of sexual selection pressure for size exaggeration via formant lowering [9], rather than precursors to speech-like abilities as once presumed. Other probable adaptations for size exaggeration include tracheal elongation in more than sixty bird species, the function of which remained a mystery for centuries before the source-filter theory was introduced to bioacoustics [20]. Air sacs in nonhuman primates including gorillas and howler monkeys also act as resonance chambers, sometimes inflatable, and may likewise function to exaggerate body size by lowering formants [56, 57].

Formants have been central in comparative research on the origins of speech and precursors of articulation, focusing heavily but not exclusively on primates. Understanding why the larynx is positioned lower in the vocal tract of humans compared to other primates, or why air sacs and vocal membranes were lost in the hominin lineage, requires first understanding the functions of these divergent anatomical adaptations. Researchers have traditionally hypothesized that selection pressure for speech intelligibility, such as a broader vowel range allotted by a longer vocal tract, or clearer articulation due to the absence of air sacs [58] and/or vocal membranes [59], explains why air sacs and vocal membranes are present in other primates but were lost during human evolution. But the exclusivity of such speech-centered explanations has been called into question in light of comparative data and emerging research findings. For example, a descended larynx may not be necessary for some animals to produce contrasting vowel patterns [60, 61], including non-uniform formant shifts observed in Diana monkey alarm calls [62]. Conversely, the descended larynx was once thought to be uniquely human but has now been observed in a wide range of mammals including deer, lions, koalas, and seals, who lack spoken language (reviewed in [9]). In these phylogenetically diverse species, and possibly in adult human males [63], the descended larynx may have little to do with speech and more to do with formant modulation for size exaggeration. Deceptive manipulation of formant frequencies has also been postulated to play a role in the evolution of vocal control, a necessary prerequisite of speech [64]. Finally, changes in facial morphology tied to posture, feeding, or prey capture might have side effects on larynx position which in turn effect vocal acoustics (e.g., shortening of the facial skeleton may “push” the larynx lower [65, 66]).

Taken together, these examples illustrate how source-filter theory places the vocal apparatus, which in most vertebrates is largely hidden, at the center of vocal communication. Selection pressures affect the morphology and control of the vocal apparatus rather than the acoustic signal itself. We thus cannot understand the signal independently of vocal morphology and its neural control. Indeed, by grounding vocal signals in their physiological mechanisms of production, the source-filter framework has offered bioacousticians increased predictive and explanatory power. The study of specific vocal features by researchers is now guided by knowledge about their biomechanical origin, their likely covariation with biological and behavioral traits, and thus their likely information content and function. Paired with recent advances in digital technologies, this enables researchers to conceptualize and answer critical questions that previously could not be empirically investigated. For example, the function of formant frequencies as cues to size in the sexual calls of terrestrial mammals could not be considered before it was realized that the bands of energy in these calls correspond to vocal tract resonances. Today, much of the acoustic diversity of vertebrate vocal signals can be interpreted in light of selective pressures affecting specific acoustic features within production constraints. In short, while the study of formants finds its roots in speech science, later generalizations of source-filter theory to non-human vocalizations revolutionized animal communication research.

In a pleasing historical turn-about, applications of this powerful theory have more recently returned full circle to our own species in the context of human nonlinguistic vocal signals, significantly advancing our understanding of how the human voice has been shaped by selection to encode (and exaggerate) biologically and socially relevant information about speakers, and how this may have paved the way for speech [64]. Empowered by the source-filter framework and discoveries of vocal production mechanisms in vertebrates, researchers have begun to investigate previously ignored aspects of human vocal behavior, shifting the focus from human speech production to nonverbal communication. Notably, voice scientists have turned their attention to questions about the evolutionary origins of human vocal signals: how have selection pressures shaped the underlying acoustic features of the human voice, including formants?

Early work at the turn of the century, which paralleled the source-filter revolution in bioacoustics, centered on static vocal indices of speaker traits in the context of human sexual selection. These studies showed that individual differences in *f*_o_ and formants can function as indices of numerous biologically and socially relevant traits such as dominance [67, 68], masculinity [55, 69], body size [28, 70, 71], and attractiveness or mate quality [72–74], wherein information about such traits is encoded in the acoustic signal itself and can be reliably decoded by human listeners. Indeed, perception experiments show that *f*_o_ and formants predict listeners’ perceptions of these and many other speaker traits, with important evolutionary and social implications (for review see [75]). Although the fundamental frequency is highly salient in the human voice and plays a key role in influencing listeners’ perceptions of multiple speaker traits (see [68, 75] for reviews), formants also predict perceptions of traits such as body size, masculinity, and attractiveness, especially when experimentally manipulated to be as perceptually salient as pitch [55]. It has become increasingly clear that, at least in human mate choice and intrasexual competition, often what matters is not *what* you say but *how* you say it.

More recent work in the human voice sciences has focused on the importance of modulating nonverbal vocal parameters for potential social and fitness benefits (see [76, 77] for reviews). In this context of deceptive signaling [78], source-filter theory has again provided critical insight into questions such as why people round their lips to sound more masculine [69, 79] or speak with lower, more closely spaced formants in contexts of authority [80] or to sound larger [63]. This emerging research field largely supports the hypothesis that selection has favored dynamic modulation of formants and other vocal parameters that exaggerate or maximize fitness-related or socially beneficial traits. A growing number of studies have also shown that human voice modulation is prevalent not only in speech and singing [81], but also during the production of nonverbal vocalizations such as cries, screams, and laughter (see [81–83]). While cries, screams, and laughter-like play vocalizations are in fact shared across many species [84], humans are particularly adept at volitionally modulating the source-filter properties of nonverbal vocalizations or producing them completely on demand, often for social benefits [64, 84, 85].

In summary, understanding the acoustic principles and bio-mechanical constraints on acoustic parameters including formants, and the costs and benefits associated with their production or modulation, has allowed researchers to uncover their honest and deceptive functions in both human and non-human animals.

### Pitfalls

We now turn to potential pitfalls in formant research. Because research on formants originated in the speech sciences, methods for analyzing formants were likewise originally designed for human speech. As we have seen, the source-filter theory describes voice production not only in humans but in most terrestrial vertebrates [7]. These methods are therefore applicable in principle to the vocalizations of other mammals, amphibians, reptiles, and birds. However, most tools for formant analysis remain optimized for human voices, which means that they typically cannot be correctly applied “off the shelf” to analyze the vocalizations of another species.

An even more fundamental concern is that formant tracking is inherently noisy, and it is harder to detect and correct measurement errors in non-human species because in most cases we lack the kind of deep insight into their vocal production that has been generated by decades of voice research in humans.

The most common method used for measuring formant frequencies and bandwidths is Linear Predictive Coding (LPC). Conceptually, LPC offers a way to fit a smooth spectral envelope under the assumption that the filter consists of a specific, user-selected number of formants (a so-called all-pole model) [86]. Default parameters such as the number of poles are typically pre-set for human adults, and for animals need to be manually adjusted based on visual inspection of formant tracks and prior knowledge of vocal production when using interactive voice analysis programs such as *Praat* [87]. Running a standard script on a large collection of audio files without manual verification of formant tracks produces errors, particularly when the audio quality is poor. Furthermore, the output of human-optimized LPC becomes progressively meaningless as we move further away from human-sized vocal tracts to very small (e.g., mouse) or very large (e.g., elephant) animals, if the algorithm is run using default parameters, unadjusted to the species in question. Nevertheless, it is still common to find uncritically accepted automatic LPC measurements of formant frequencies—and even of their bandwidths—using standard phonetic software with default parameters for sounds very far removed from the vocal tract configurations and frequency ranges of human vowels, such as macaque and bonobo calls [88] or violin music [89].

**Fig. 2:**
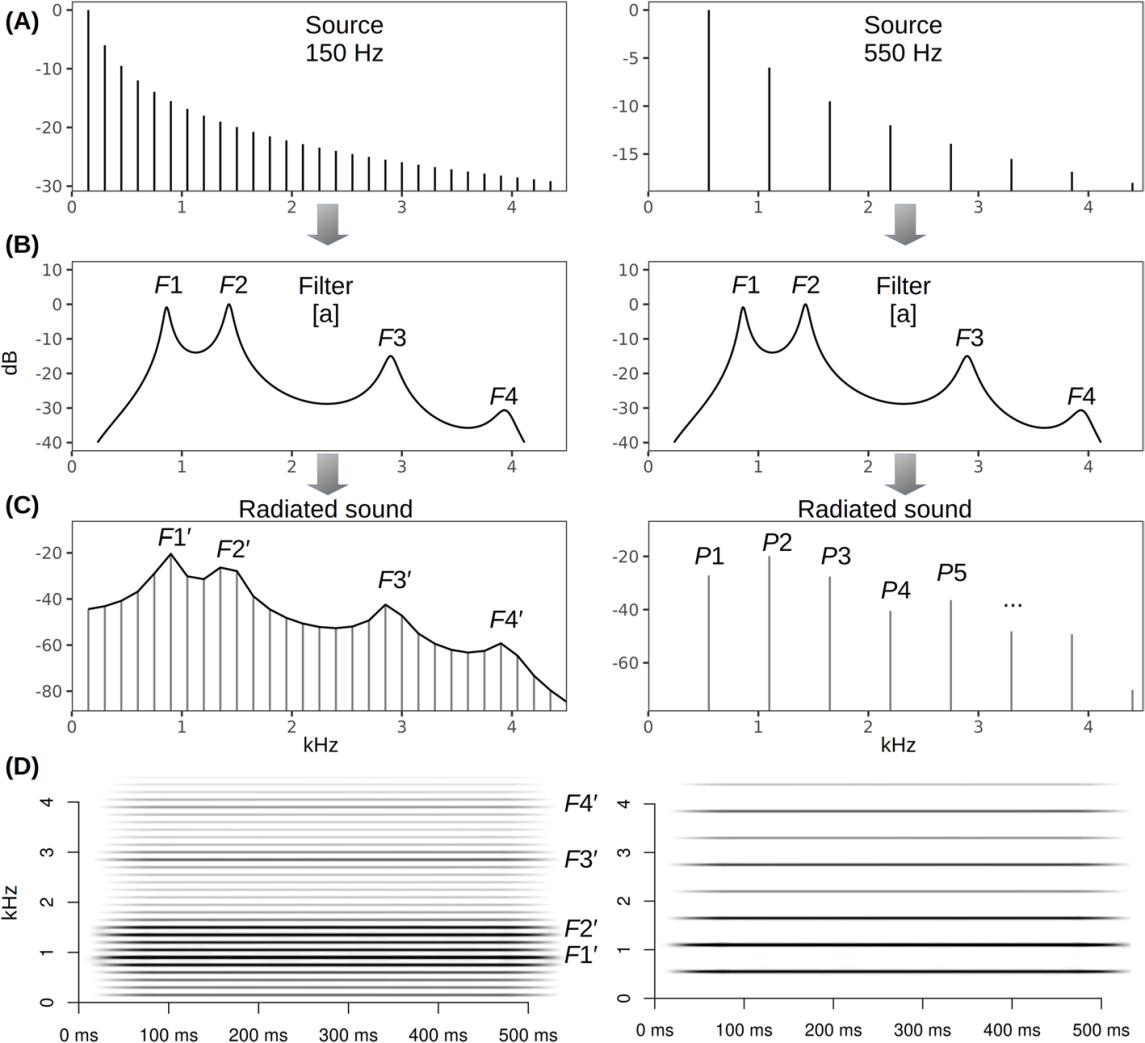
Formant under-sampling by a high-*f*_o_ source. **A** Top panels: Two tonal sounds with *f*_o_ of 150 Hz (left) and 550 Hz (right) are filtered by **B** middle panels: the same transfer function corresponding to vowel [a] spoken by a person with a 15.7 cm long vocal tract (*c* = 354 m/s). **C **and** D **(left panels): dense harmonics of the 150 Hz vowel clearly reveal spectral peaks—putative formants *F*1′ to *F*4′ that closely correspond to the true vocal tract resonance frequencies. In contrast (**C **and** D**; right panels), in the vocal signal with *f*_o_ of 550 Hz, we also observe spectral peaks (*P*1, *P*2, …); but these now correspond to harmonics of *f*_o_, deviate considerably from the true vocal tract resonances, and should not be confused with formants. Note that although the fundamental frequency is typically partial with the highest amplitude in the initial source signal, higher harmonics may be stronger in the output signal after filtering. Diagnostics: harmonics are always spaced at exact integer multiples of *f*_o_, whereas formants can vary independently of one another and thus are rarely perfectly evenly spaced

Proper use of LPC with animal vocalizations requires, at a minimum, first deriving an estimate of vocal tract length using whatever data are available (e.g. measurements on museum skulls, x-rays, or even a photograph of the animal’s head with an object of known length to provide scaling). Then, assuming a cylindrical vocal tract of this length, formant frequencies can be predicted using Eq. 1, and this information can be used to choose the appropriate number of poles for formant analysis in LPC (with two poles per formant) [90, 91]. When reporting these data in published research, we suggest that the estimated vocal tract length should always be provided, along with the LPC parameters used, in order to increase transparency and replicability in formant research [3]. In addition to adjustments based on the length of the vocal tract, LPC is highly sensitive to source characteristics. Formant analysis in voiced speech is only tractable when *f*_o_ is considerably lower than *F*1, but even so, LPC estimates are biased towards nearby harmonics, while the formants perceived by human listeners are much closer to the true resonance frequencies [92]. The higher the *f*_o_, the more likely it is that LPC will track individual harmonics rather than vocal tract resonances. This problem is obvious when *f*_o_ is actually higher than *F*1, such that the first resonance is not excited at all by energy from the source. But formants may become “invisible” even when *f*_o_ is lower than *F*1. For example, in the right panel of Fig. 2, the *f*_o_ is 550 Hz and *F*1 is 860 Hz, but the spectrum is still not dense enough to resolve the formants. This is a very real problem: there have been instances in which harmonics were erroneously reported as formants, for example, in mouse vocalizations [93, 94] or high-pitched screams of chimpanzees [95] (but see correction [96]).

Terminological confusion does not help. The fundamental frequency is often designated “*f*-zero” (*f*_o_) and formants start with “*F*-one” (*F*1) [97]. Perhaps owing to this notational similarity, the fundamental frequency itself is sometimes erroneously referred to as a formant, for instance: “Tongue and jaw position serve to change the configuration of the vocal tract and affect which frequencies will resonate most strongly. The lowest of these formants (i.e., fundamental frequency) corresponds with the pitch of a vowel” [98]. In our opinion, it is erroneous to refer to spectral peaks as “formants” if they are simply partials of the produced tone (*f*_o_ or one of its harmonics) because these frequencies are entirely determined by the voice source, and not by the resonances of the vocal tract filter [99]. Similarly, the frequency with the highest amplitude (“dominant frequency”) in a filtered sound could represent *f*_o_, one of its higher harmonics (e.g., 2*f_o_ or 3*f_o_, as in Fig. 2F), or a formant frequency excited by noise [100, 101], and confusing them will lead to errors. Avoiding such errors is important because all harmonics in a voiced sound are tightly coupled, while true formants can be flexibly modified independently of one another. Thus, if the two are confused or conflated it could lead to massive underestimations of vocal tract flexibility in the species in question.

**Fig. 3:**
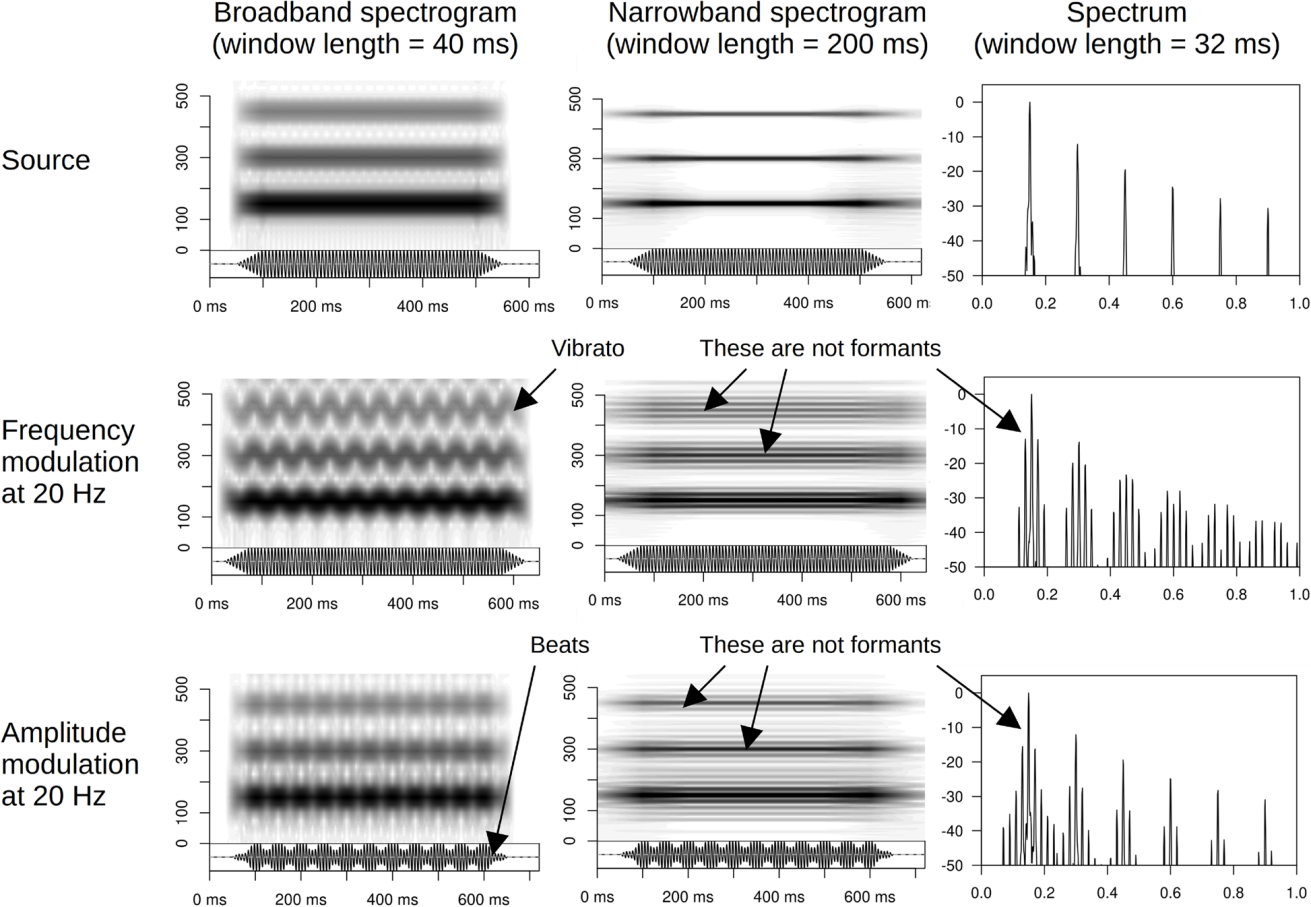
Pseudo-formants caused by frequency modulation or amplitude modulation. A tonal sound at an *f*_o_ of 150 Hz is modulated at 20 Hz. Both frequency modulation (vibrato one semitone in depth) and amplitude modulation (non-sinusoidal oscillator at half the amplitude of the carrier wave) produce sidebands around harmonics, which can resemble formants in the spectrum or on narrowband spectrograms. Diagnostics: modulation can typically be perceived by ear. Frequency modulation (FM) can be detected visually in the broadband spectrogram as vibrato-like frequency oscillation at 20 Hz, especially in the upper harmonics, while amplitude modulation (AM) produces noticeable beats at 20 Hz in the oscillogram (bottom panel beneath each spectrogram)

Considering the well-known limitations of LPC, it is standard practice to recommend manual inspection of spectrograms as a final “sanity check” of automatic formant measurements. Enhanced visual representations, such as reassigned spectrograms [92, 102], also provide promising new approaches to measuring formants, which may avoid some problems with LPC. However, while we agree that manual checking is important, it is crucial to emphasize that visual detection of formants is also far from trivial. With non-linguistic human and animal vocalizations in particular, source modulation and/or nonlinear acoustic phenomena (Table 1) may create spectral peaks that superficially resemble formants but have nothing to do with the vocal tract filter. For example, periodic modulations of *f*_o_ known as frequency modulation (FM) are easy to hear and visualize as such when they are slow (e.g., under 10 Hz in opera-style vibrato). However, rapid FM of the kind found in ultrasonic vocalizations of rodents [48], dog whines [103], or some songbirds [104, 105] produces sidebands corresponding to new frequency components around each harmonic of *f*_o_ (Fig. 3). Likewise, amplitude modulation (AM) of the main source frequency by other oscillators, such as respiratory variability in human vibrato singing or budgerigar contact calls [106, 107], intralaryngeal oscillations of the arytenoids in toads [108], or vibration of the ventricular folds in some styles of rock singing [109], will generate sidebands around the harmonics. In both FM and AM, the spacing of these sidebands is equal to the modulation frequency. If *f*_o_ is relatively high, and the modulation rate low, these sidebands or “pseudo-formants” can easily be mistaken for formants (see Fig. 3).

**Fig. 4:**
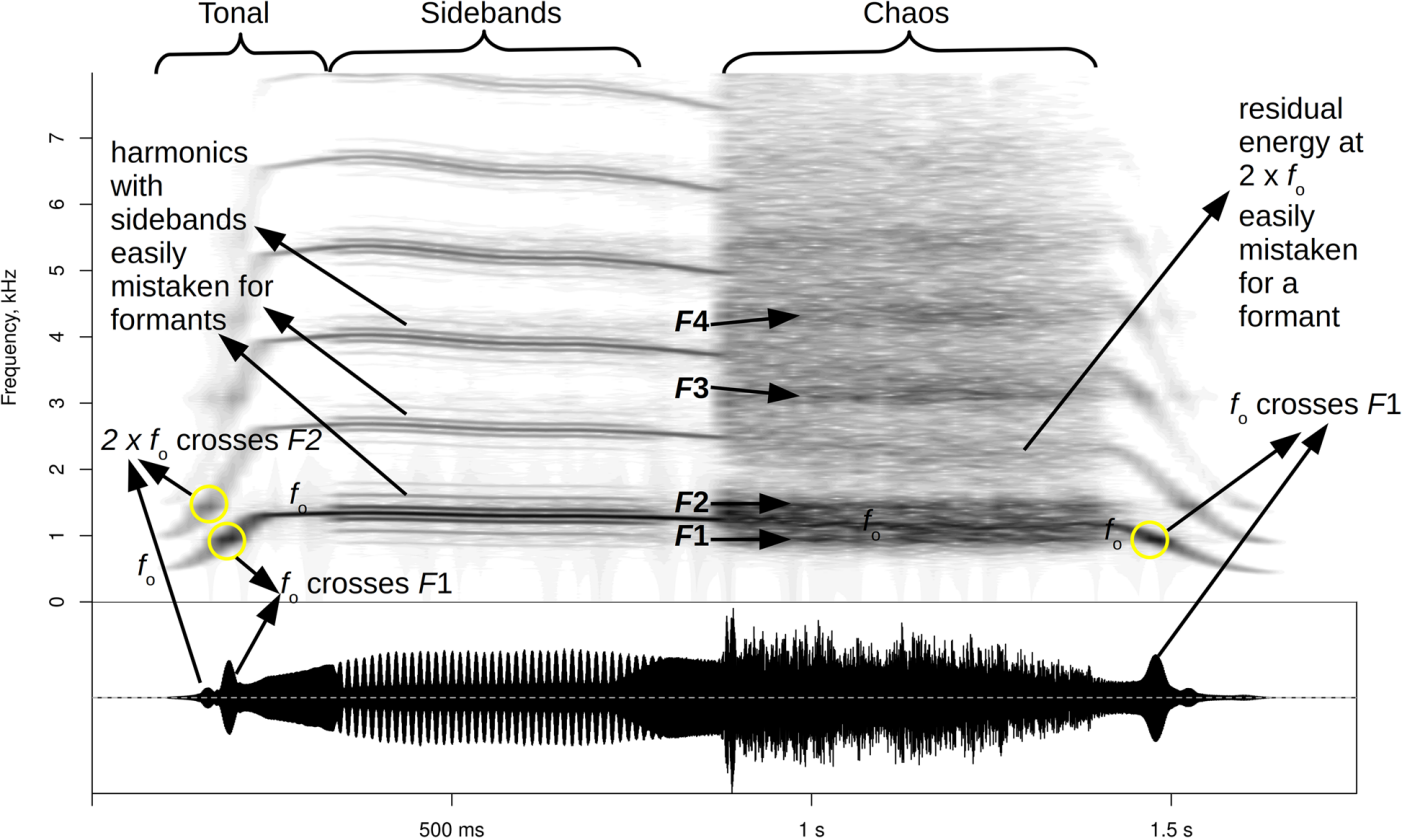
Pitfalls and specialized opportunities for formant tracking in high-pitched vocalizations. The formant structure is virtually invisible in the tonal part of this modulated high-pitched call, apart from slight changes in amplitude as *f*_o_ crosses the first formant (yellow circles). Nonlinear phenomena (see Table 1) may help to reveal formant frequencies (here, “true” formants, labeled *F*1 to *F*4, are visible in the chaos), but caution is needed to avoid confusing formants with sidebands (resulting from modulation) or residual harmonics

In addition to sidebands, many animal vocalizations contain voiced, but very noisy or practically atonal episodes of deterministic chaos, which is the most perceptually salient type of nonlinear acoustic phenomenon (e.g., common in monkey screams, dog barks, and deer roars [110], human cries, screams and roars [81], some frog vocalizations [111, 112], and even some fish vocalizations [113]. Due to its broad-band nature, chaos may actually help delineate otherwise invisible formants in high-pitched calls like chimpanzee pant-hoots or human screams, but extreme caution is needed because source harmonics may persist but become blurred, turning them into broad, formant-like spectral peaks (Fig. 4). A safer strategy is to look for call sections that contain other broadband noise such as respiration or vocal fry with individual glottal cycles separated by long silence (e.g., in fallow deer groans [114]. It is also sometimes possible to detect formants indirectly, if *f*_o_ varies, from minor changes in the amplitude of individual harmonics as they cross a formant (Fig. 4, yellow circles). Finally, source-filter interactions in the form of formant locking may create sudden frequency jumps from one formant to the next, such that formant frequencies can be estimated simply from discrete values of *f*_o_ [38, 115]. However, such specialized techniques of formant tracking are specific to particular call types in particular species, typically cannot be automated, and require considerable prior insight into vocal production in the analyzed species.

Finally, while most investigations of formant frequencies assume that vocal tracts consist of a single tube closed at the glottis and open at the lips (Fig. 1), one must consider that in some species, the vocal tract often incorporates side branches, including the nasal cavity in vertebrates or air sacs in non-human primates and other mammals. Calls can be nasal only, oral only, or simultaneously oral and nasal, and additional branches can be opened or closed at the velar junction of the oral and nasal cavities. A study by Reby et al. [116] showed that formant patterns observed in fallow deer groans are better predicted by vocal tract geometry that considers both the oral and nasal airways, as indicated by CT-imaging of the vocal tract in dead specimens positioned in a calling posture (stretched neck and retracted larynx). Vocal tracts can also include air sacs, acting as side branches of the vocal tract, and often with characteristics of a Helmholtz resonator [56, 61, 117]. Such complex geometries are typically associated with additional formants [118] and thus complicate the prediction of the number of poles in LPC (or related parameters in other analysis methods, such as the smoothing factor in cepstral smoothing). In calls with long wide-open glottal phases, the glottis might need to be modeled as open (thus deviating from Eq. 1), and tracheal resonances may be involved. Generally, as these examples effectively illustrate, it is critical to consider the anatomical mechanisms of vocal production in order to properly adjust the parameters of formant analyses for non-human animal calls.

### The promise of formant analysis in bioacoustics

Assuming that the pitfalls described above can be avoided, research on formants in animal vocalizations can be both scientifically sound and biologically illuminating and shows considerable promise. As we have seen, because the source-filter theory and methodology for formant analysis was developed largely in the context of human phonetic research, this framework typically requires adaptation before it can be applied to non-human animals.

Birds provide several nice examples. In about half of extant species (the oscine songbirds), the avian syrinx is a doubled organ, and the two sides are capable of producing two independent frequencies (a “two-voiced” dual source, and thus biphonation: [105, 119, 120]. However, in some cases these two sources may be coupled, yielding FM and sidebands and thus potential "pseudoformants" by the principles described above [104].

Furthermore, when considering the signaling role of formants, clade-specific anatomy must be taken into account. For example, because the main determinant of formants, vocal tract length, typically correlates with body size, formants provide “honest” cues to the vocalizer’s size in a wide range of tetrapods, including alligators and many mammals [9, 17, 18]. However, in birds, the syringeal sound source in all birds rests at the base of the trachea, so avian vocal tract length includes tracheal length [20]. This means that birds will typically have much longer vocal tracts, and lower formants, than other tetrapods of comparable size. This anatomical difference also explains why tracheal elongation, a putative size-exaggerating trait, has evolved in over 60 species of bird but in no other vertebrates [20].

A second challenge in cross-species comparisons involves accounting for differences in vocal tract morphology and body or vocal tract size across species, requiring some form of vocalizer-specific normalization. In some studies, the impression of size is the variable of interest (e.g., to see if formants provide accurate cues to body size), and normalization may be omitted. However, much recent research focuses on the degree to which animals can modify vocal tract shape to achieve a variety of formant patterns, reminiscent of a human vowel space [60, 61]. Because smaller animals with shorter vocal tract lengths have higher formants, the same proportional change in shape would lead to much larger absolute frequency changes compared to a larger animal. In such cases, normalization is required.

**Fig. 5:**
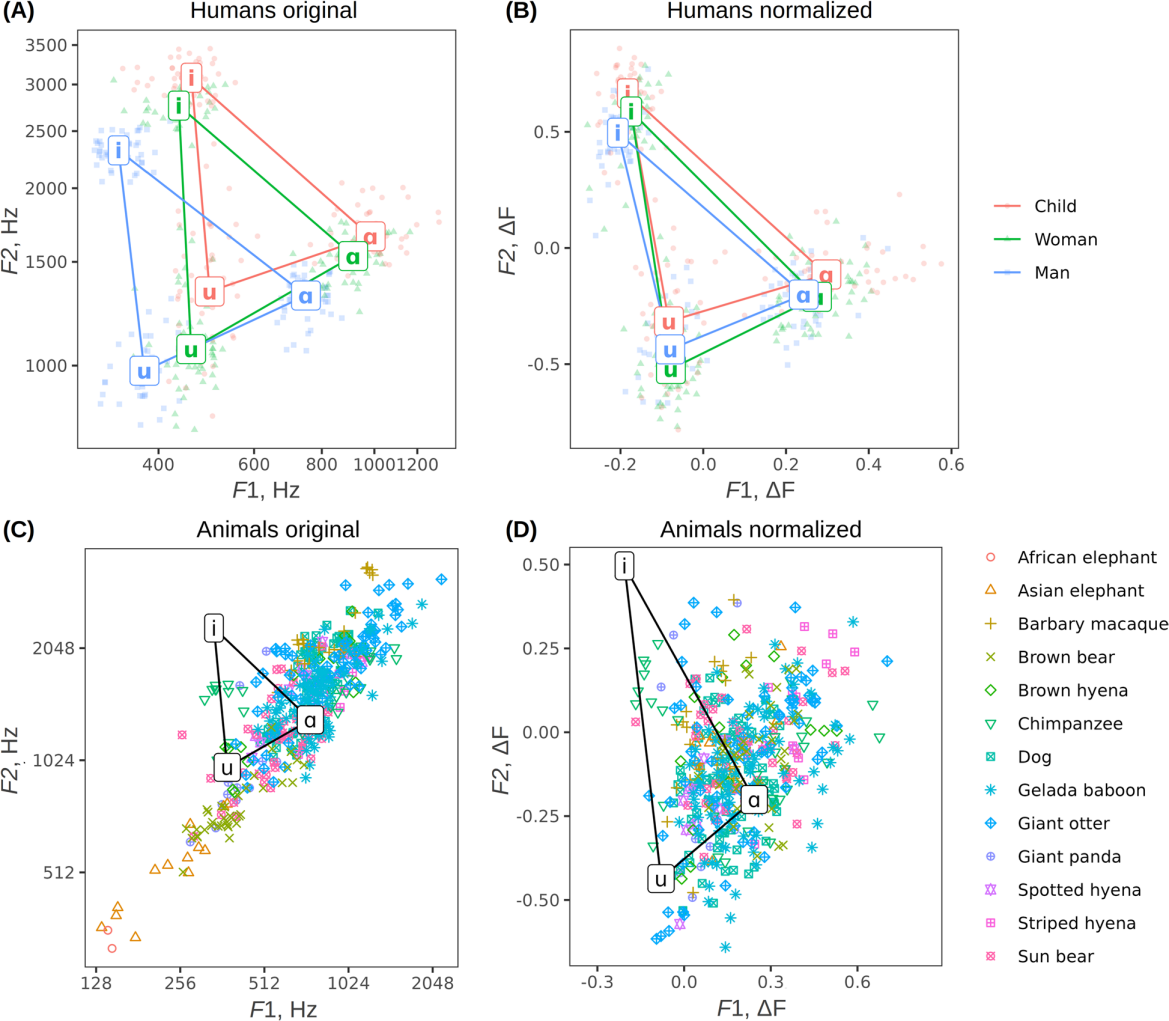
Speaker normalization applied to human and nonhuman mammal vocalizations. **A** Human formant measurements in Hz vary greatly across speakers, producing three different vowel spaces for adult men, adult women, and children. **B** Normalization: In contrast, vowel spaces become more similar after formants in Hertz are normalized to vocal tract length by recalculating to formant spacing (ΔF) units above or below the expected neutral frequencies in a relaxed vocal tract whose length is estimated from the original formant measurements [122]. **C** “Raw” formant measurements of vowel-like calls of species greatly varying in size are too species-specific to allow valid direct comparisons of formant space. **D** Normalization: these calls can be projected onto a shared normalized vowel space. *N* = 1668 human vowels in panels **A**–**B** [123] and 457 non-human animal calls from 13 species in panels **C**–**D** (authors’ data)

The simplest computational model for vocalizer-specific normalization divides all observed formant frequencies by the same estimated speaker-specific scaling constant, while more sophisticated methods perform regression on one or more vowels from the same speaker. This approach, until now most commonly applied to human speech [121, 122], can be easily extended to animal vocalizations (see below). For example, when working with a single vowel-like vocalization recorded from an animal, a simple normalization technique is based on estimating vocal tract length from the original formant measurements and prior knowledge of the animal’s vocal anatomy or body size at the species level, calculating theoretically predicted formant frequencies in a cylindrical vocal tract of this length, and then comparing the observed to predicted formant frequencies (Fig. 5A–B). As a result, absolute formant measurements in Hz, which are incommensurable across animals of such different sizes as mice, monkeys, and elephants, can be converted into relative measurements of how high or low each formant is relative to its neutral position. This can be mapped onto an *F*1/*F*2 space, and interpreted as vowel quality, regardless of the size of the species, and can even be directly compared with the human vowel space [122]; see Fig. 5C–D).

One advantage of this two-dimensional, normalized formant (*F*1/*F*2) space is that, in conjunction with hypotheses pertaining to the perceptual effects of formants, predictions can be made about the distribution of call types in this space according to their potential function. There is growing evidence that, just as in human speech, formant patterns in vertebrate vocalizations often depart from the even spacing corresponding to a relaxed vocal tract, indicating some level of articulatory manipulation (see, e.g., Fig. 2 in [64]). While the presence of vocal tract control does not appear to correlate with nascent linguistic abilities, understanding the adaptive functions of articulatory perturbations may shed light on why basic articulatory abilities evolved that could later have been co-opted for speech production in the human lineage [64].

**Fig. 6:**
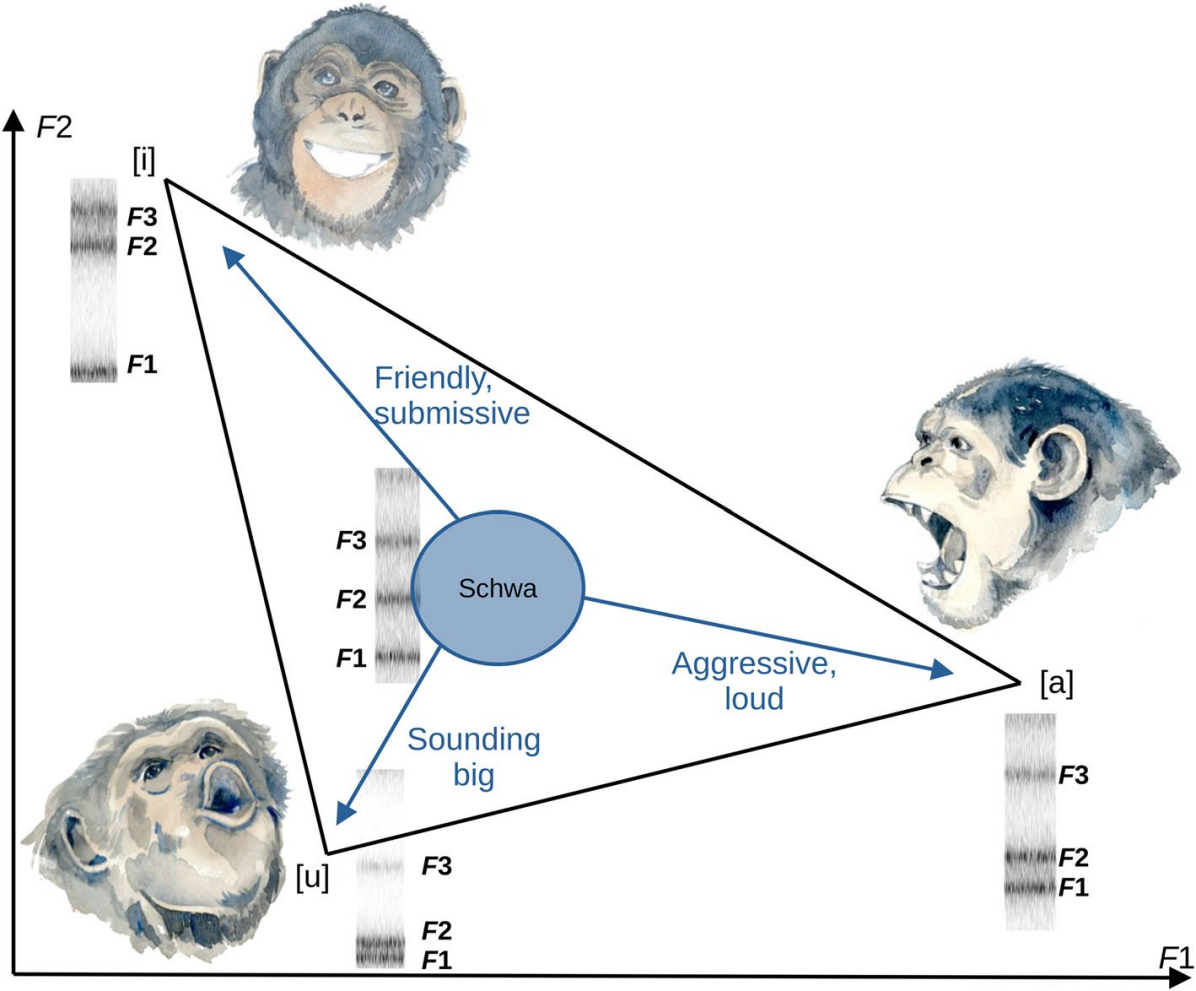
Predicted changes in vowel quality of animal calls depending on the context, relative to an unarticulated schwa vowel [ə]. Based on what we know about sound symbolism in human vocalizations and speech, and recent research on vocal strategies for size exaggeration or intimidation [124, 125], we predict that formant spacing corresponding to [i]-like vowels in vocalizations should be optimal for conveying submission or friendly intentions (sounding small and harmless), whereas [a] should be optimal for long-distance or aggressive calls, and [u] for size exaggeration

As already mentioned, a prominent potential example of an adaptive function of vocal tract manipulation in animals is provided by size exaggeration. If shifting individual formants (as in human vowels) has similar perceptual effects as scaling all formants down equally (by increasing vocal tract length), callers might capitalize on this bias to offer an alternative route to achieving size exaggeration. Indeed, there is some evidence from experiments involving human listeners to support this: lowering one or two formants in human or animal-like vocal signals appears to have the same perceptual effect as scaling the entire vocal tract, making the vocalizer sound bigger [124]. Whether animals capitalize on this perceptual bias in real-world contexts remains to be investigated. Similarly, we predict that affiliative calls may not only be produced by a short vocal tract (spread lips and/or raised larynx) but may also predominantly contain formant patterns with a relatively high *F*2 as in the vowel [i]. In contrast, aggressive calls may not only be produced with a long vocal tract (lowered larynx and/or rounded lips), but also with a lowered *F*2 as in the vowel [u] (see Fig. 6). While this may affect within-call-type variation along the affective dimensions of valence and arousal, we also predict that it will be reflected in formant distributions across the different call types that compose vocal repertoires. Plotting formants measured in submissive versus aggressive calls from multiple species in normalized *F*1/*F*2 space, as proposed above (Fig. 5), will allow this hypothesis to be tested.

Importantly, just as under-sampling of the vocal tract filter by a high-frequency source is an issue for measuring formants as discussed above (see Fig 2), it is also an issue for perceiving them. If the periodicity of a non-noisy source is too high relative to the spacing between formants in that call, then formants are not excited or resolved and are unlikely to have any strong perceptual relevance [9, 34]. We thus predict that calls selected to communicate socially or biologically important information through formant frequencies should be characterized by a dense source spectrum so that formant frequencies are perceptually salient. This could be achieved by vocalizing with a relatively low *f*_o_, as demonstrated with low-pitched speech in humans [35, 126]. It may also be achieved via low-pitched growls or by producing broadband noise with or without phonation, as in roars and hisses, respectively. This constraint should be particularly relevant in calls with formants that are relatively low (narrowly spaced) or unevenly distributed (with two formants spaced close to one another as in [i]), as well as in smaller animals with higher *f*_o_. In some cases, including in the calls of young individuals in many mammal species, formant frequencies may be highlighted in relatively high-pitched vocalizations by means of adding vibrato [9, 36].

At the same time, retaining a relatively high fundamental frequency may sometimes be desirable. This is because producing an *f*_o_ lower than one’s modal or baseline *f*_o_ (as predicted from vocal *fold* length) involves a decrease in efficiency, and thus can come at the expense of voice intensity or loudness. Indeed, new evidence suggests that acoustic intensity is important not only to ensure sufficient sound propagation [127], but also to convey aggressive intent and demonstrate physical prowess in confrontational contexts [125]. Because *f*_o_ and *F*1 both covary with voice intensity, there exists a major trade-off between low frequency and loudness in human vocal production, with only the most formidable of individuals able to maintain a low pitch while vocalizing loudly [125]. This trade-off may be resolved by a diversification of call types (e.g., Iberian deer [128]), by combinations of “syllables” (wa-hoo in baboons [129]), or by biphonation (wapiti [115], horses [130]). Thus, we can expect diversity in vocal repertoires to evolve in response to constraints that result, either directly or indirectly, from the basic principles of vocal production we have discussed here.

While, with a few possible exceptions (elephant trumpet calls, aerodynamic whistles), source and filter can typically be assumed to be independent, we also predict that the interplay between the source and filter will affect the acoustic structure of calls. Aligning the source periodicity with formant patterns (formant tuning) may boost amplitude, as seen in both human soprano singing [131] and in gibbon loud calls [43] and it is likely that formant tuning is common in loud calls in many other species [132]. It is also possible that more complex calls can be produced by combining “normal” voiced sources with aerodynamic whistles. This “whistle hypothesis” could explain the prominent high-frequency, nearly pure-tone second frequency visible in biphonated wapiti roars or horse whinnies [115, 130]. The manner in which the high-frequency component "hops" from one vocal tract resonance to another in wapiti bugles is consistent with this whistle hypothesis. Heliox experiments would allow this hypothesis to be directly tested.

More generally, a range of unexplained morphological adaptations in the vocal production system, from syringeal bullae in ducks to zygomatic pouches in paca, remain little-studied, but can likely be understood based on the source/filter principles reviewed here [133]. We strongly suspect that evolution has “tinkered” with vocal production acoustics across many species to make their vocal output more diverse or more impressive to conspecific listeners, and that such added complexity can yield fitness benefits to the vocalizer.

## Conclusion

In the first part of this review, we explained how the acoustic and physiological principles of vocal production and specifically source-filter theory, originally developed for human speech, have recently been extended to nonhuman vertebrates, leading to novel interpretations and a richer understanding of animal communication systems. However, these principles must be understood, and typically adjusted, before they can be appropriately applied to analyses of the vocalizations of a chosen nonhuman animal species. If this is not done (e.g., by utilizing automatic acoustic analysis routines intended for human speech, without modification), significant errors can result. We have shown how this can, and does, easily occur, particularly in the analysis of formant frequencies in animal calls. We have also highlighted that not all call types will be suited for all analysis types, another potential source of errors or confounds in bioacoustics. 

When these perils are avoided, research in the last two decades demonstrates the power of the source-filter theory: equipped with an understanding of vocal production, researchers can gain rich insights into the evolution of communication and of vocal repertoires, in a remarkable diversity of species from frogs and birds to bats and whales. Furthermore, particularly in the context of primate communication, bioacoustically informed comparative research on formants can offer deep insights into the evolution of vocal communication in our own species, including both speech and singing, but also the human nonverbal vocal repertoire that includes laughter, cries, screams, groans and roars. We conclude that, when fully understood and appropriately applied, the acoustic principles of vocal production provide access to exciting and still largely unexplored avenues for future research, promising to enrich and deepen our understanding of vertebrate vocal communication and its evolution.

## Data Availability

No datasets were generated or analysed during the current study.
